# Abnormally increased DNA methylation in chorionic tissue might play an important role in development of ectopic pregnancy

**DOI:** 10.1186/s12958-021-00785-2

**Published:** 2021-07-02

**Authors:** Wen Cai, Liu Yang, Ruiqing Zhang, Yixia Yang, Shuangdi Li, Jiarong Zhang

**Affiliations:** 1 Department of Obstetris and Gynecology, Shanghai General Hospital, School of Medicine, Shanghai Jiao Tong University, Shanghai, China; 2 Department of Obstetris and Gynecology, Zhongshan Hospital Fudan University, Shanghai, China; 3 Shanghai First Maternity and Infant Hospital, Shanghai, China

**Keywords:** DNA methylation, Ectopic pregnancy, Intrauterine pregnancy, Chorionic tissue, Bioinformatics

## Abstract

**Background:**

Human Ectopic Pregnancy (hEP) is the second most common cause of pregnancy-related deaths in the first trimester. Without timely detection, EPs can lead to an increased rate of infertility and an elevated risk for future tubal EPs. In addition, most studies in the field focus on the effect of the fallopian tube (maternal factors) and ignore epigenetic changes in genes and proteins of the embryo, which may also cause EPs. Therefore, the present study hypothesized that embryos also play an important role in the development of EP. The study also speculated that DNA methylation is associated with ectopic pregnancy. Consequently, the effects of DNA methylation on the occurrence and development of ectopic pregnancy were investigated. Moreover, genome-wide DNA methylation of chorionic tissue from ectopic and intrauterine pregnancies was detected using Illumina HumanMethylation450 arrays.

**Results:**

Forty-three hypermethylated genes involved in the regulation of adhesion as well as gene transcription and translation were identified. Furthermore, the PPI network showed that AMOTL1, SDR42E1, CAMTA1, PIP5K1C, KIAA1614, TSTD1 and DNER may play important roles in the occurrence and development of ectopic pregnancy. In addition, SDR42E1, CAMTA1 and TSTD1 displayed higher levels of methylation in ectopic pregnancy while PIP5K1C and DNER showed low degrees of methylation.

**Conclusions:**

The study reveals that abnormal increase in methylation may be an early indicator or an inducer of ectopic pregnancy. In addition, AMOTL1, SDR42E1, CAMTA1, PIP5K1C, KIAA1614, TSTD1 and DNER might play important roles in the occurrence and development of ectopic pregnancy. However, the specific molecular mechanisms are still unclear and require further studies.

**Supplementary Information:**

The online version contains supplementary material available at 10.1186/s12958-021-00785-2.

## Introduction

Human ectopic pregnancy (hEP) is the second most common cause of pregnancy-related deaths in the first trimester [1, 2], and approximately 98% of EPs occur in the fallopian tube [3]. Without timely detection, EPs can lead to an increased rate of infertility and an elevated risk of future tubal EPs [2]. The fallopian tube plays an important role in transporting the female and male gametes, in which fertilization, early embryonic development, and transport of the embryo to the uterus take place [4]. It is generally believed that epigenetic changes in genes and proteins of the fallopian tube lead to alterations in the microenvironment of fertilized eggs as well as their implantation hence leading to the occurrence of EPs [5]. In normal case, the egg and sperm meet and fertilize in the fallopian tube. After 3–4 days, they wander into the uterine cavity, where the zona pellucida undergo structural changes and embryo implant. Additionally, blastocyst implantation can be divided into three steps: localization, adhesion and invasion. Notably, EPs occur mainly due to errors in localization and adhesion despite the lack of animal models. Because fertilized eggs cannot be used as experimental materials, research in the area remains largely scarce. Currently, ectopic pregnancy is mainly believed to occur due to the presence of blastocysts in the fallopian tube. Furthermore, the abnormal peristalsis of the fallopian tube caused by tubal inflammation and the fact that blastocyst development is not synchronized with the endometrium (implantation stage), are considered to be highly risky. However, most studies focus on the effect of the fallopian tube (maternal factors) and ignore epigenetic changes in genes and proteins of the embryo, which may also cause EPs. Therefore, the present study hypothesized that embryos also play an important role in the development of EPs. For example, the rapid replication and division of blastocyst cells and the disappearance of the premature zona pellucida may lead to implantation in the fallopian tube. In addition, imprinting genes may cause abnormal blastocyst development or make the movement of blastocyst sticky.

During reproduction, epigenetic modification occurs at two key periods including gamete development and trophoblast cell growth before implantation [6]. Epigenetics refers to changes in the expression level and function of genes without changes in the DNA sequences, resulting in a heritable phenotype. Therefore, abnormal expression of these epigenetic-regulated genes may lead to failure in normal implantation and placental abnormalities, eventually causing anomalies in fetal growth or preeclampsia. Furthermore, DNA methylation is one of the main methods of epigenetic modification. It involves the addition of a methyl group to the carbon at position 5 of the cytosine pyrimidine ring or the nitrogen at position 6 of the adenine purine ring through the catalysis of DNA Methyltransferases (DNMTs) and plays an important role in normal development [7]. In humans, DNA methylation only occurs on cytosine bases and results in the formation of 5-methylcytosine (5-mC), which can regulate genomic activity and can be maintained throughout mitosis and meiosis [8]. Moreover, the addition of methyl groups changes the biophysical characteristics of the DNA, alters binding of transcription factors and other chromatin interacting proteins, chromatin structure and accessibility, thus fine-tuning gene expression [7]. More evidence suggests that epigenetic mechanisms, including DNA methylation, are involved in the regulation of endometrial changes during the menstrual cycle [9–11], the implantation process [12, 13], and early embryo development [14]. In addition, DNMTs are essential for normal embryo development. For instance, Saitou et al. reported that Dnmt1-3a/b-deficient mice are associated with increased embryonic lethality or death after birth [14]. Several clinical studies have shown that epigenetic changes are often associated with adverse pregnancy outcomes [15]. According to a recent report on the global DNA methylation status in normal human fallopian tubes, aberrant DNA methylation was shown to be present when the fallopian tube became dysfunctional [16]. In order to find out whether abnormal DNA methylation was present in the embryonic tissue of EP patients, we analyzed the DNA methylation status of chorionic tissues in ectopic pregnancies and 40-day intrauterine pregnancies. The results can hint on the factors affecting the embryo itself in ectopic pregnancy.

## Materials & methods

### Sample selection

This study was approved by the Institutional Review Boards of Shanghai Jiaotong University (2018KY170). Participants were recruited from the Department of Obstetrics and Gynecology, Shanghai General Hospital, following diagnosis of ectopic pregnancy. Informed consent was obtained from each participant. All participants meet the following requirements: younger than 35 years old, with normal vital organ functions and metabolic functions, and no acute or chronic diseases. We selected chorionic tissues after operations, of which 6 were from women with ectopic pregnancy and 6 were from women with intrauterine pregnancy. All samples were stored in liquid nitrogen immediately.

### Genome-wide DNA methylation analysis

Genomic DNA from the samples was bisulfite treated as the protocol of Zymo EZ DNA Methylation kit (Zymo Research, www.zymoresearch.com). DNA methylation was then analyzed using the Infinium HD Methylation450K BeadChip Array (Illumina, San Diego, CA, USA) following the standard Infinium HD Methylation450K Assay protocol.

All samples were run on the array together within the same batch and passed quality control. Data pre-processing such as background subtraction and normalization to internal array controls was performed in GenomeStudio® Methylation Module v1.8 Methylation Module 1.9 software (http://www.illumina.com).

### CpG probe filtering

The 485,577 methylation probes from the Human Methylation450 array were filtered. The DNA methylation was measured and with detection *p* values above the significance threshold 0.05.

Microarray chips were scanned by a HiScan 2000 (Illumina). To minimize any effects of sample processing, validation cohort arrays were run in the same batch and with the same operators as a subset of the samples from the discovery cohort. This DNA methylation data for the discovery and validation cohorts is available from the Gene Expression Omnibus (GEO) database under the accession numbers GSE100197 and GSE98224, respectively.

Raw data (IDAT Files) were read into R statistical software, version 3.2.4, where functional normalization, background subtraction, and color correction were performed as Blair et al. previously used subset within-array normalization (SWAN). Functional normalization performs all the benefits of SWAN normalization and, in addition, utilizes the 900 control probes on the array to mediate changes in DNA methylation that are due to technical effects [17].

### Functional enrichment analysis

To identify prominent clinical mechanisms affecting and/or affected by growth restriction in our cohort, the 172 genes with evidence of differential methylation were investigated with respect to their functions and major biological roles. The analysis was performed using DAVID [18]. All *p* values were FDR corrected. The 296 putatively affected CpG set was also analyzed in the context of wider genomic regions using GREAT [19]. We set the distal extension to the nearest genes as 100 kbp. The background set of probes to which the comparison was made was defined as the 399,118 autosomal CpGs used as the initial input to our analysis pipeline.

### PPI network analysis

WGCNA package was used for co-expression analysis of DEGs and then the data has been superimposed onto the PPI database of STRING [20]. The co-expression analysis clusters were delineated by the dynamic tree cut package with the minimum height for each module at 0.2 [21]. The consensus trend of each module was based upon eigengene, and the members of module were collected by Pearson correlation among DEGs and their interactors. Moreover, a topological overlapping matrix was also utilized to filter the PPI network [22].

### Bisulfite sequencing PCR

When genomic DNA is modified with sodium bisulfite, all the unmethylated Cytosine (C) is converted to Uracil (U), while methylated cytosine remains unchanged. After treating genomic DNA with sulfite, the study designed the corresponding BSP primers to amplify the target fragment. At this time, all the Uracil (U) molecules had been converted to Thymine (T) and the PCR product was finally sequenced to determine whether the CpG site was methylated.

## Results

The study sought to identify novel candidate genes associated with EPs by comparing genome-wide DNA methylation using Illumina HumanMethylation450 arrays of chorionic tissues from ectopic and intrauterine pregnancies.

### Widespread DNA methylation changes are associated with EPs in chorionic tissues

**Fig. 1:**
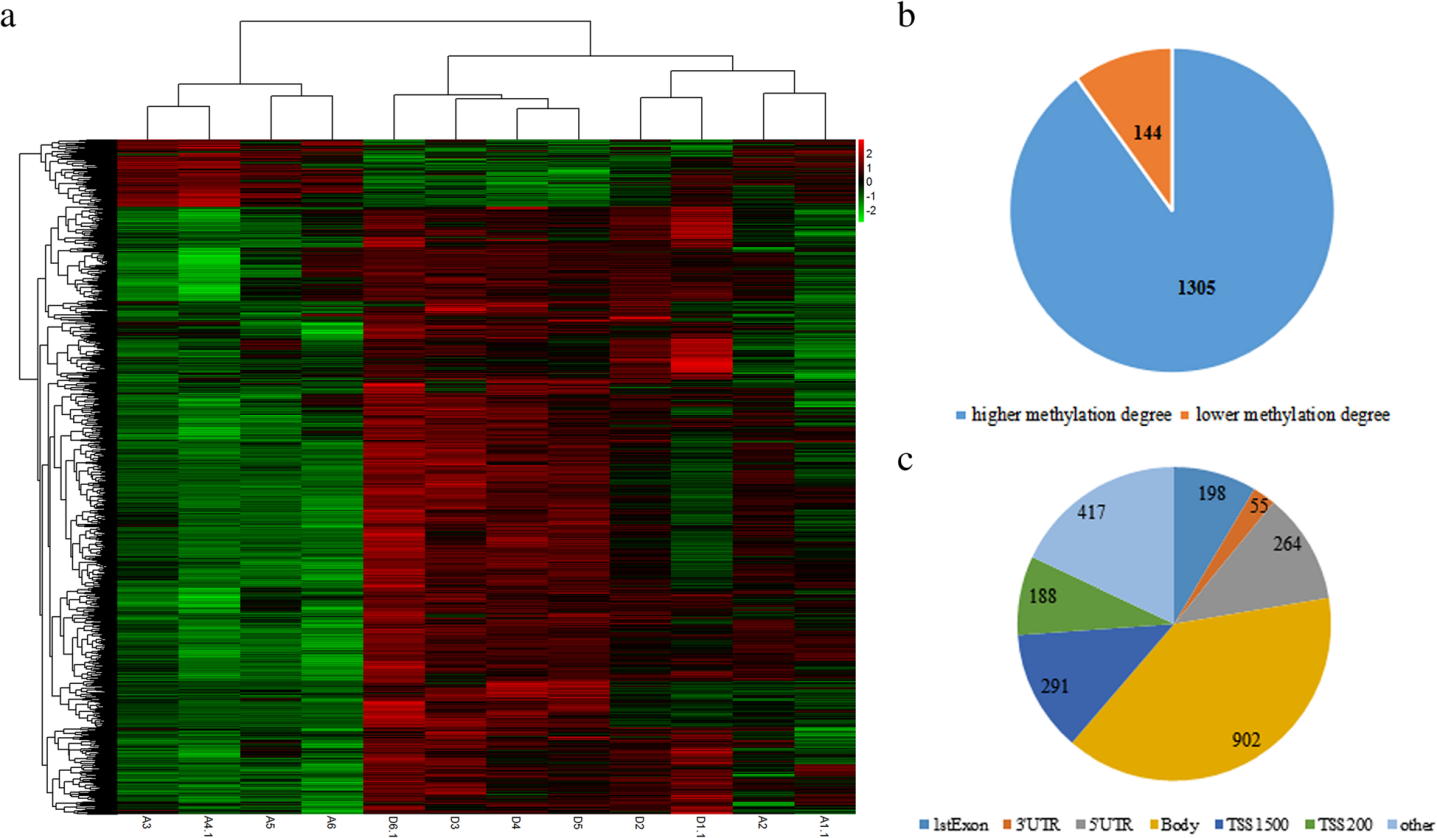
Differential Methylation Cluster Analysis in chorionic tissue from ectopic pregnancy and intrauterine pregnancy. **a** A Heatmap of Differential Methylation in chorionic tissue from ectopic pregnancy and intrauterine pregnancy (A1.1, A2, A3, A4.1, A5 and A6 are from intrauterine pregnancy, and D1.1, D2, D3, D4, D5 and D6.1 are from ectopic pregnancy). **b** 1449 differential methylated genes were obtained, which include 1305 significantly hypermethylated genes 144 significantly hypomethylated genes; **c** T differential methylation sites occurring in 1stExon, 3’UTR, 5’UTR, Body, TSS1500, TSS200, Other were 198, 55, 264, 902, 291, 188, 417 respectively

Differential DNA methylation analysis was performed on a total of 485,577 sites, using these CpGs to quantify the methylation profiles resulted in the EP in chorionic tissue from ectopic pregnancy and intrauterine pregnancy (Fig. 1A). This suggested that the identified collection of CpGs is sufficiently representative of methylation differences in EP. As showed in Fig. 1B, compared with the intrauterine pregnancy, 1449 differentially methylated sites were screened out in the ectopic pregnancy group, differential methylation sites occurring in 1stExon, 3’UTR, 5’UTR, Body, TSS1500, TSS200, Other were 198, 55, 264, 902, 291, 188, 417 respectively (Fig. 1C). There were 1305 sites with higher methylation level and 144 sites with lower methylation level. The average methylation level of all the difference sites in the ectopic pregnancy group was higher than that in the intrauterine pregnancy group, and the difference was statistically significant (*P* < 0.05, Fig. S1).

### Functional enrichment

**Fig. 2:**
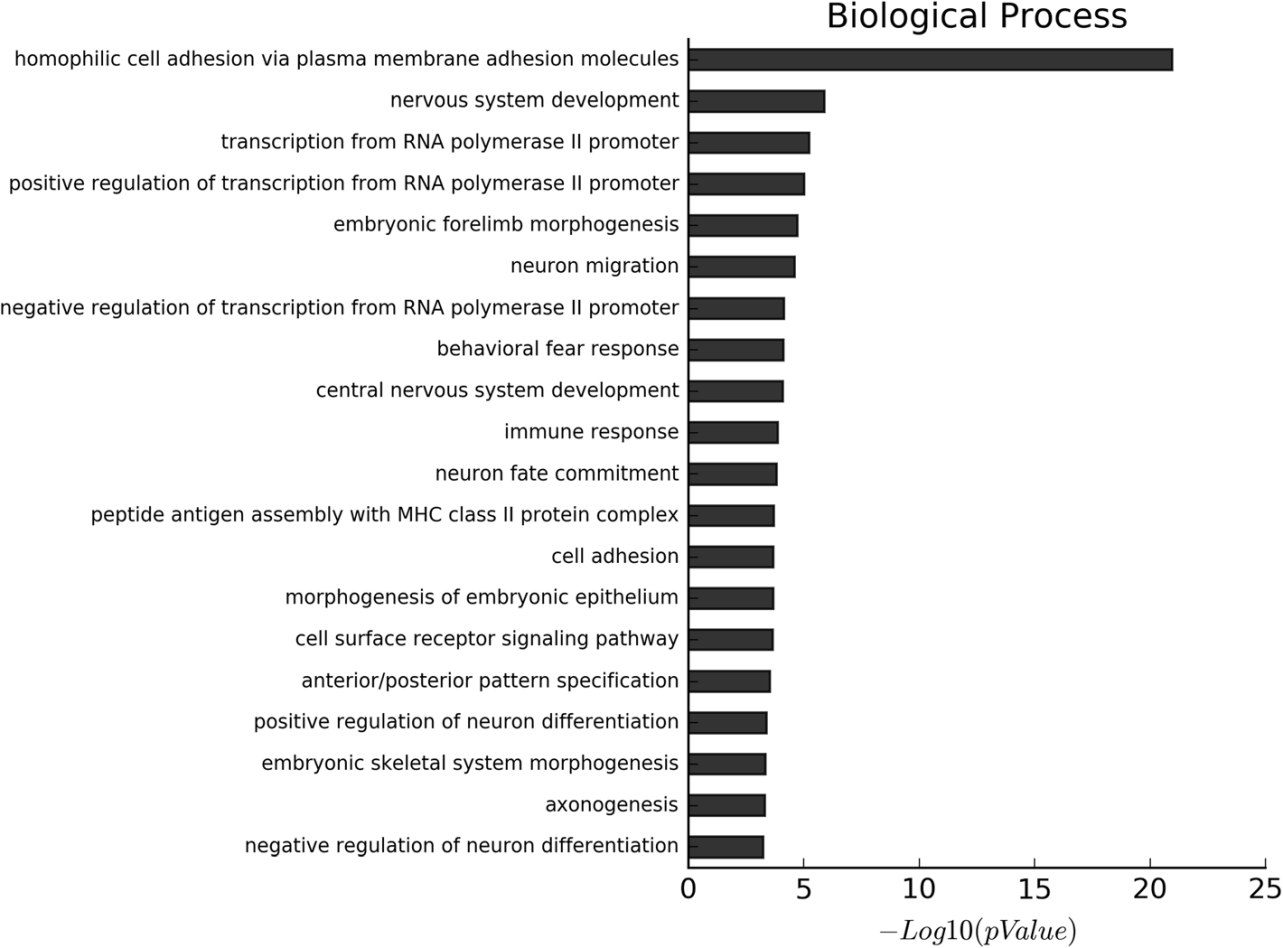
The biological process GO analysis of all differential methylated genes: The most enriched GO targets were involved in homophilic cell adhesion via plasma membrane adhesion molecules, nervous system development, transcription from RNA polymerase II promoter et al

**Fig. 3:**
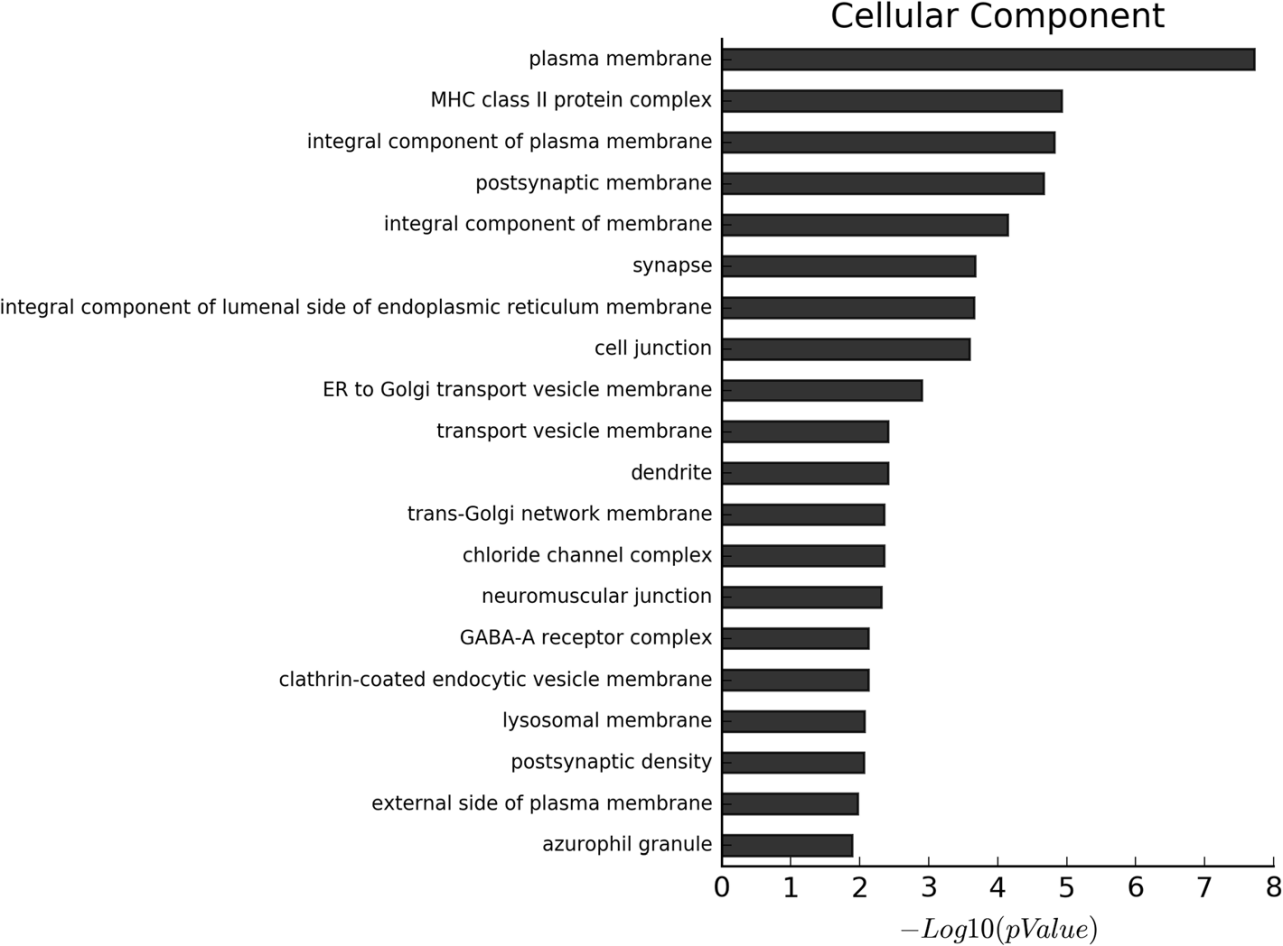
The cellular component GO analysis of all differential methylated genes: The most enriched GO targets were involved in plasma membrane, MHC class II protein complex, integral component of plasma membrane et al

**Fig. 4:**
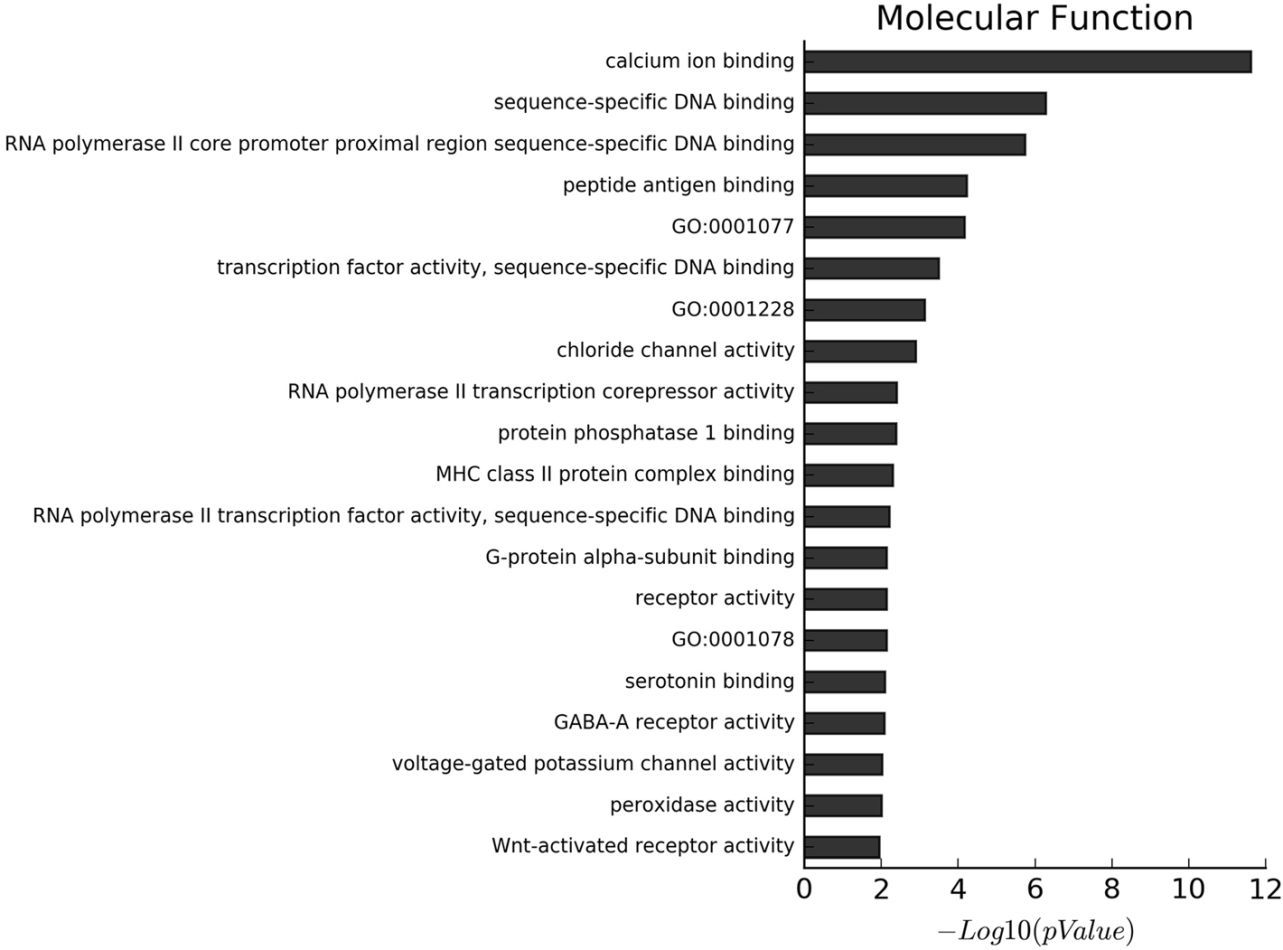
The molecular function GO analysis of all differential methylated genes: The most enriched GO targets were involved in calcium ion binding, sequence-specific DNA binding, RNA polymerase II core promoter proximal region sequence-specific DNA binding et al

**Fig. 5:**
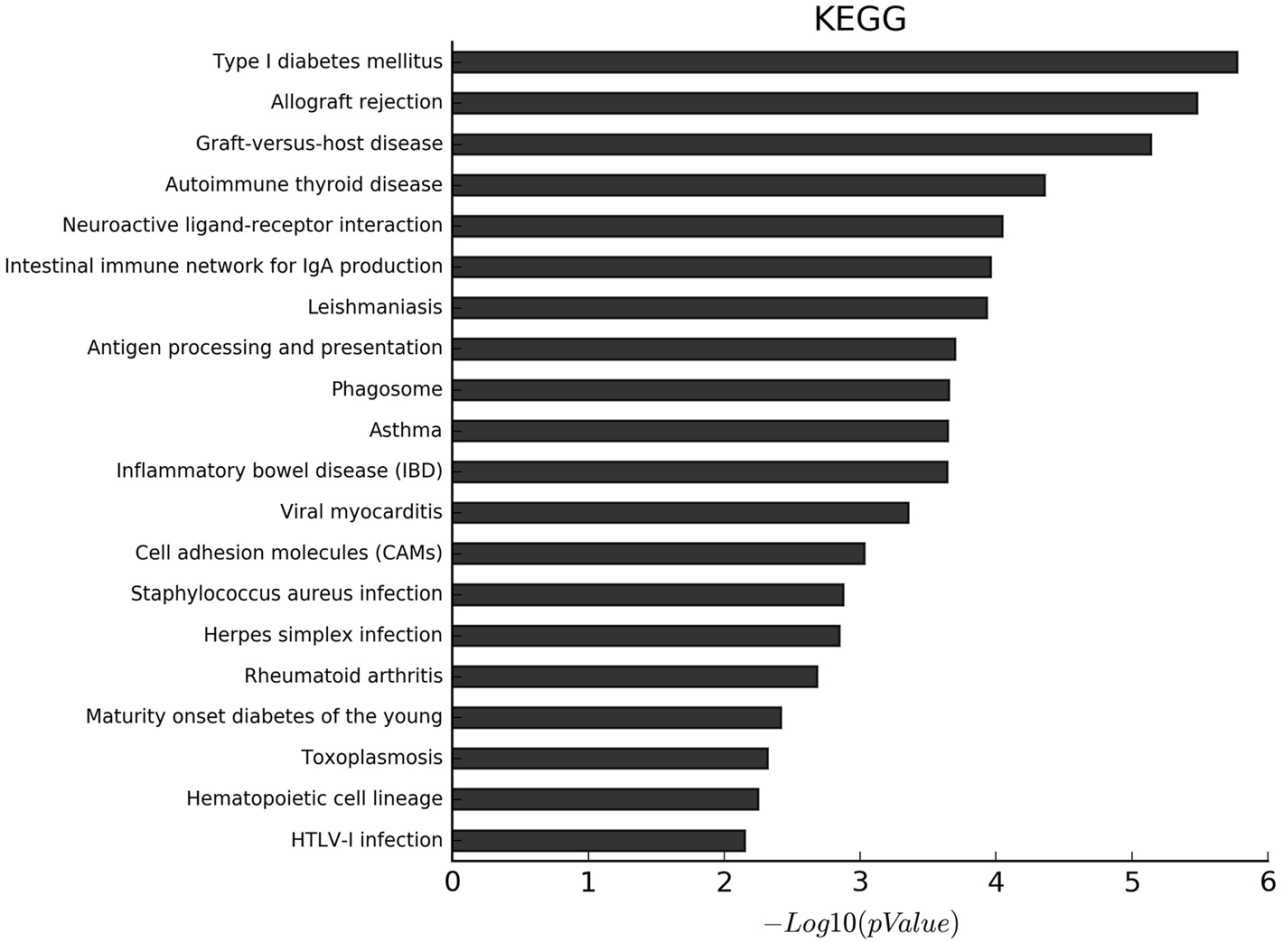
The KEGG analysis of all differential methylated genes: The most enriched KEGG targets were involved in Type 1 diabetes mellitus, allograft rejection, graft-versus-host disease, autoimmune thyroid disease et al

GO and KEGG enrichment analyses showed a significant over-representation of functional groups highly relevant to the EP phenotype. The proportion of genes with increased levels of methylation accounted for about 90% of the differentially methylated genes. Additionally, the results of GO analysis of the biological process of all the differentially methylated genes, hypermethylated genes and hypomethylated genes, are shown in Fig. 2, Fig. S2 and Fig. S3, respectively. On the other hand, the results of GO analysis of the cellular component of all the differentially methylated genes, hypermethylated genes and hypomethylated genes, are shown in Fig. 3, Fig. S4, and Fig. S5, respectively. Moreover, the results of GO analysis of the molecular function of the hypermethylated genes and hypomethylated genes are in Fig. 4, Fig. S6 and Fig. S7. Furthermore, the enriched functional groups were related to calcium ion binding, sequence-specific DNA binding, RNA polymerase II core promoter proximal region sequence-specific DNA binding. In addition, the results of KEGG analysis of all the differentially methylated genes, hypermethylated genes and hypomethylated genes, are shown in Fig. 5, Fig. S8 and Fig. S9, respectively. According to the results, the enriched functional groups were related to Type 1 diabetes mellitus, allograft rejection, graft-versus-host disease and autoimmune thyroid disease.

The study further analyzed 43 genes (Table 1) with large differences in methylation. The results in Table 2 showed that PARVG, PIP5K1C and TGFB1I1 were involved in focal adhesion while IL18RAP, STX2, UMODL1, CD34, SDR42E1, PTPRN2, XKR5, ANO1, F2RL1, ROBO3 and EMP3 were linked to the integral component of membrane. On the other hand, PARVG, IL18RAP, STX2, CD34, ANO1, F2RL1, EMP3 and RHOF were associated with the plasma membrane while PARVG, TGFB1I1 and RHOF were involved in the cytoskeleton. Additionally, CAMTA1, BCL11A, F2RL1, HOXD13 and LMX1A were involved in positive regulation of transcription from the RNA polymerase II promoter while ADAMTS14, PAPLN, TGFB1I1 and LMX1A were linked to zinc ion binding. Moreover, ZNF823, BCL11A, CAND2 and LMX1A were involved in transcription, DNA-templated while ZNF823, HOXD13 and LMX1A were associated with DNA binding. Finally, CAMTA1, ZNF823, BCL11A, CAND2, HOXD13, PIP5K1C, LMX1A and MAPKAPK2 were involved in the nucleus. The results therefore suggested that the 43 genes (Table 1) with large differences in methylation, were mostly involved in the regulation of cell adhesion as well as gene transcription and translation.

**Table 1:** Genes of significant differences in the degree of methylation between ectopic pregnancy group and intrauterine pregnancy group

Gene Name	DiffScore	Gene Group	Gene Name	DiffScore	Gene Group
ROBO3	−30.29132	Body	EMP3	−35.37979	3’UTR
SDR42E1	−51.20948	TSS200	PIP5K1C	−34.52377	Body
FBXL18	−37.65086	Body	IL18RAP	−33.66035	5’UTR
VPS53	−30.68286	Body	PAPLN	−30.91774	TSS1500
RHOF	−32.21111	Body	F2RL1	−31.40948	Body
PTPRN2	−31.92761	Body	SARM1	−30.32454	Body
CD34	−38.95479	TSS1500	UMODL1	−32.76879	Body
LMX1A	−33.11592	Body	BCL11A	−31.41892	Body
TGFB1I1	−35.75037	Body	ZNF823	−32.49986	Body
AMOTL1	− 46.43716	Body	PIP5K1C	−30.28541	Body
NXPH4	−33.79389	Body	HHIPL1	−34.38662	Body
LOC151174	−32.38859	Body;TSS1500	HOXD13	−32.92324	TSS1500
MAPKAPK2	−34.36791	Body	PIP5K1C	−41.7007	Body
CAMTA1	−47.58438	Body	MIAT	−34.51908	Body
KIAA1614	−34.03587	TSS1500	KIAA1614	−42.41064	TSS1500
PIP5K1C	−34.03009	Body	ADAMTS14	−31.65536	Body
BCL11A	−38.03922	Body	FAIM3	−30.86871	5’UTR;1stExon
CAMTA1	−37.85619	Body	ANO1	−30.67168	Body
STX2	−35.49095	Body	PTPRN2	−30.11571	Body
PARVG	−34.85364	5’UTR;Body;1stExon	CAND2	−30.67898	Body
XKR5	−31.15694	TSS1500	TSTD1	−52.06646	5’UTR;1stExon
DNER	34.68416	Body

**Table 2:** GO enrichment analysis of 43 differential genes (partial)

Category	Functions	%	*P*-value	Genes
CC	focal adhesion	8.9	0.128	PARVG, PIP5K1C, TGFB1I1
CC	integral component of membrane	32.4	0.291	IL18RAP, STX2, UMODL1, CD34, SDR42E1, PTPRN2, XKR5, ANO1, F2RL1, ROBO3, EMP3
CC	plasma membrane	23.5	0.494	PARVG, IL18RAP, STX2, CD34, ANO1, F2RL1, EMP3, RHOF
CC	cytoskeleton	8.8	0.117	PARVG, TGFB1I1, RHOF
BP	positive regulation of transcription from RNA polymerase II promoter	14.7	0.070	CAMTA1, BCL11A, F2RL1, HOXD13, LMX1A,
MF	zinc ion binding	11.8	0.267	ADAMTS14, PAPLN, TGFB1I1, LMX1A
BP	transcription, DNA-templated	11.8	0.623	ZNF823, BCL11A, CAND2, LMX1A
MF	DNA binding	8.8	0.745	ZNF823, HOXD13, LMX1A
CC	nucleus	23.5	0.803	CAMTA1, ZNF823, BCL11A, CAND2, HOXD13, PIP5K1C, LMX1A, MAPKAPK2

### PPI network analysis of genes with the greatest differences in the level of methylation

**Fig. 6:**
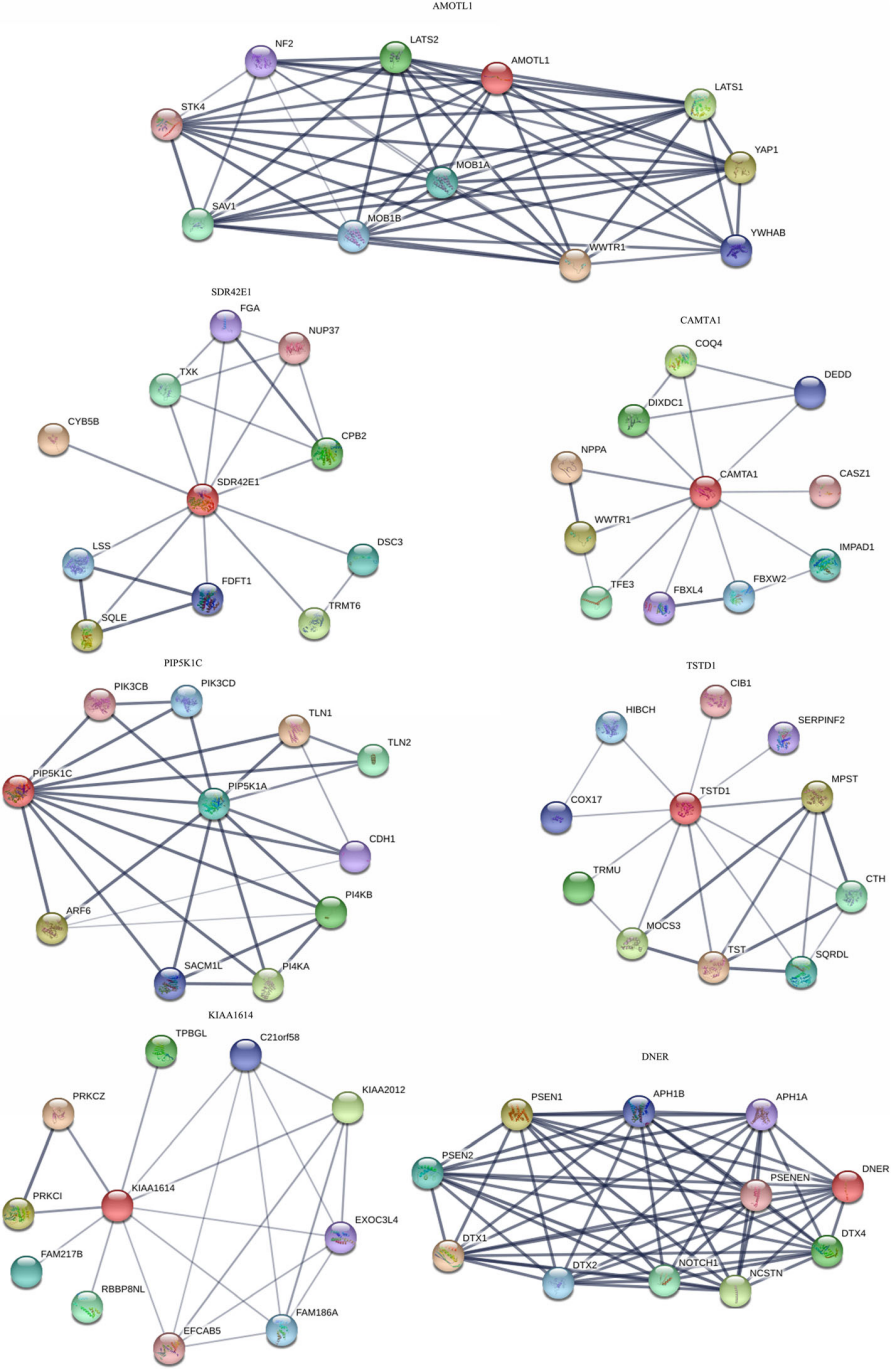
PPI network analysis of genes with the greatest difference in methylation level: Those genes were AMOTL1, SDR42E1, CAMTA1, PIP5K1C, KIAA1614, TSTD1 and DNER

The study then analyzed the PPI network of 7 genes with the greatest differences in methylation. The genes included; AMOTL1, SDR42E1, CAMTA1, PIP5K1C, KIAA1614, TSTD1 and DNER (Fig. 6). Notably, AMOTL1 belongs to the angiomotin family and plays an important role in the migration and polarization of endothelial cells [23–25]. It can also inhibit the Wnt/beta-catenin signaling pathway, probably by recruiting CTNNB1 to recycling endosomes, hence preventing its translocation to the nucleus [26]. In addition, abnormal methylation might inhibit the migration of blastocysts, ultimately leading to their retention in the fallopian tube. Additionally, SDR42E1 was shown to be the source of oxidoreductase activity and is involved in the steroid biosynthetic process. On the other hand, CAMTA1 has the DNA-binding transcription factor activity and is involved in the regulation of transcription.

PIP5K1C catalyzes the phosphorylation of Phosphatidylinositol 4-phosphate (PtdIns4P) to form phosphatidylinositol 4, 5-bisphosphate (PtdIns (4, 5) P2). It also participates in a variety of cellular processes, including vesicle mediated transport, cell adhesion, cell polarization, cell migration and cell junction assembly. In addition, it modulates the formation of adhesion junctions by facilitating CDH1 trafficking and also regulates the targeting of talins (TLN1 and TLN2) to the plasma membrane as well as their efficient assembly into focal adhesions. In addition, the gene regulates the interaction between talins (TLN1 and TLN2) and beta-integrins and is required for the formation of uropodium as well as retraction of the cell rear during directed migration. It also has a role in growth factor-stimulated directional cell migration and adhesion. Moreover, it is required for the assembly of talins into nascent adhesions forming at the leading edge towards the direction of the growth factor, and it is a negative regulator of T-cell activation and adhesion. It also negatively regulates the polarization and adhesion of integrin alpha-L/beta-2 (LFA-1), induced by the T-cell receptor. Together with PIP5K1A and PIP5K1B, it has a role in embryogenesis and may play a role immediately after birth [27–30]. Furthermore, TSTD1 has thiosulfate-thiol sulfurtransferase activity. Sulfurtransferase (TST) is required to produce S-sulfanylglutathione (GSS-), which is a central intermediate in hydrogen sulfide metabolism. It also provides the link between the first steps in mammalian H2S metabolism performed by the sulfide, quinone oxidoreductase (SQOR) which catalyzes the conversion of H_2_S to thiosulfate and sulfur dioxygenase (SDO), which uses GSS- as a substrate. Additionally, the thermodynamic coupling of the irreversible SDO and reversible TST reactions provides a model for the physiologically relevant reaction with thiosulfate as the sulfane donor [31]. Finally, DNER is an activator of the NOTCH1 pathway, which may mediate neuron-glia interaction during astrocytogenesis.

### Bisulfite sequencing PCR analysis of 6 genes with the greatest differences in the levels of methylation

Six genes with the greatest differences in methylation were selected to conduct Bisulfite Sequencing PCR. The genes included, SDR42E1, CAMTA1, PIP5K1C, KIAA1614, TSTD1 and DNER (Fig. S10, S11, S12, S13, S14 and S15). The study performed Bisulfite Sequencing PCR on the CpG sites of the 6 genes to verify the level of methylation. The results revealed that SDR42E1, CAMTA1 and TSTD1 had higher levels of methylation in EPs while PIP5K1C and DNER had lower levels. In addition, the methylation changes in the 5 genes mentioned above were consistent with the detection results from the chip. However, there was no significant difference in the expression of KIAA1614 among all the samples. Nonetheless, more samples are needed for further verifications.

## Discussion

Ectopic pregnancy (EP) process in which embryos are implanted outside the uterine cavity and fallopian tube pregnancy is the most common. The occurrence of the disease is mainly due to the inflammation in or around the fallopian tube. The inflammation can make the lumen obstructed and obstruct the normal operation of the pregnant ovum, so that the embryo stays implants and develops in the fallopian tube, leading to the abortion or rupture of the fallopian tube pregnancy.

Studying the molecular mechanism of ectopic pregnancy is important for early detection and effective prevention of the condition. In addition, extensive research has shown that abnormal expression of genes and proteins as well as the function and structure of the fallopian tube are among the main factors leading to ectopic pregnancy. For example, H. Enzan et al. reported that disappearance of CD34-positive and alpha-smooth muscle actin-positive stromal cells was associated with intra-uterine and tubal pregnancies in humans [31]. Moreover, Andrew W. Horne et al. reported that changes in the expression of genes in the endometrium also play an important role in ectopic pregnancy [32]. In these previous studies, fallopian tube and endometrium related factors were collectively referred to as maternal components although the genomics and proteomics of the embryo were ignored. Notably, germ cells undergo a lot of necessary modifications in methylation during the transition from gamete formation to fertilization and embryo development [33, 34]. In this study, the results revealed extensive changes in DNA methylation between the chorionic tissue from ectopic pregnancy and that from intrauterine pregnancy. Moreover, compared to intrauterine pregnancy, 1449 differentially methylated genes were screened out in the ectopic pregnancy group. Furthermore, the 90% methylation of differentially expressed genes showed an unregulated tendency, suggesting that the abnormal elevation of methylation may be an early indicator or an inducer of ectopic pregnancy.

The study further analyzed 3 hypermethylated genes, including SDR42E1, CAMTA1 and TSTD1. SDR42E1 was shown to be a source of oxidoreductase activity and is involved in the steroid biosynthetic process. Additionally, research on embryos of mice showed that harmful free radicals may block embryo development. According to a previous study, hypermethylated SDR42E1 may affect the oxidoreductase activity and produce harmful free radicals. This may in turn not only lead to stagnation in embryonic development but may also affect the migration and adhesion of the embryo. On the other hand, members of the CAMTA family respond to Ca^2+^ signaling by binding to calmodulin [35]. Moreover, the GO terms enriched among the CAMTA1-repressed genes indicated that both mitosis and DNA replication were inhibited by CAMTA1. CAMA1 was also shown to inhibit the differentiation of tumor cells. Moreover, methylation of cytosine residues in gene-associated CpG islands was reported to be a common mechanism mediating transcriptional repression of growth-regulating genes in tumors [36]. When EP occurs, the hypermethylation of CAMA1 may inhibit embryo differentiation and affect its migratory ability. Furthermore, TSTD1 is involved in the process of hydrogen sulfide metabolism. Previous studies showed that there was in increase in abnormal H_2_S signals during ectopic pregnancy and this not only hindered transport in the fallopian tubes but also interfered with the development of embryos and the implantation process [25]. The hypermethylated TSTD1 may inhibit hydrogen sulfide metabolism, resulting to abnormally elevated H_2_S signals.

Moreover, the PIP5K1C and DNER genes which were shown to have lower levels of methylation, are also involved in many vital biological activities. For instance, PIP5K1C was shown to regulate adhesion by facilitating the activation of RhoA GTPase and integrin through chemoattractants. Additionally, Xu et al. investigated the kinase, PIP5K1C and showed that deficiency of PIP5K1C impaired neutrophil recruitment due to an adhesion defect [37]. Hypomethylation of CpG islands leads to higher levels of PIP5K1C expression, which enhances the adhesion of embryos to the fallopian tube, leading to the occurrence of EPs. On the other hand, DNER was identified as a Notch ligand that mediates cell-cell interactions [38]. In oncology, DNER was reported to execute pathological functions as both an oncogenic and anti-oncogenic factor [39]. Notably, various cell-cell interactions occur during the implantation of the embryo. However, whether DNER is also involved in these processes, remains unclear and needs to be explored further. Although there was no significant difference in methylation among the groups studied, KIAA1614 was shown to be significantly associated with the levels of circulating cellular adhesion proteins. Nonetheless, the specific molecular mechanisms need further investigations.

## Conclusion

The present study conducted an analysis of genome-wide DNA methylation of chorionic tissue from ectopic and intrauterine pregnancies using Illumina HumanMethylation450 arrays. This was done in order to examine the effect of DNA methylation on the occurrence and development of ectopic pregnancy. The results suggested that abnormal increase in methylation may be an early indicator or an inducer of ectopic pregnancy. In addition, 43 hypermethylated genes were shown to be involved in the regulation of adhesion as well as gene transcription and translation. Moreover, the PPI network showed that AMOTL1, SDR42E1, CAMTA1, PIP5K1C, KIAA1614, TSTD1 and DNER may play important roles in the occurrence and development of ectopic pregnancy. However, the specific molecular mechanisms are still unclear and require further studies. Therefore, for patients who failed to be clearly diagnosed as ectopic pregnancy by ultrasound, we can detect the methylation levels of specific genes in peripheral blood to predict ectopic pregnancy. Female descendants of patients with ectopic pregnancy can also be genetically screened to determine the risk of ectopic pregnancy. All in all, this research provides a theoretical basis for the study, early detection and effective prevention of ectopic pregnancy.

## Supplementary Information

**Additional file 1: Figure S1.** T-test of all differential methylated genes. **Figure S2.** The biological process GO analysis of hypermethylated genes. The most enriched GO targets were involved in T-helper 1 type immune response, homophilic cell adhesion via plasma membrane adhesion molecules, positive regulation of filopodium assembly et al. **Figure S3.** The biological process GO analysis of hypomethylated genes. The most enriched GO targets were involved in homophilic cell adhesion via plasma membrane adhesion molecules, transcription from RNA polymerase II promoter, nervous system development et al. **Figure S4.** The cellular component GO analysis of hypermethylated genes. The most enriched GO targets were involved in transport vesicle membrane, clathrin-coated endocytic vesicle membrane, trans-Golgi network membrane et al. **Figure S5.** The cellular component GO analysis of hypomethylated genes. The most enriched GO targets were involved in plasma membrane, integral component of plasma membrane, MHC class II protein complex et al. **Figure S6.** The molecular function GO analysis of hypermethylated genes**.** The most enriched GO targets were involved in phosphatidylinositol-3-phosphate binding, GTPase activator activity, calcium ion binding et al. **Figure S7.** The molecular function GO analysis of hypomethylated genes. The most enriched GO targets were involved in calcium binding, sequence-specific DNA binding, RNA polymerase II core promoter proximal region sequence-specific DNA binding et al. **Figure S8.** The KEGG analysis of hypermethylated genes. The most enriched KEGG targets were involved in leishmaniasis, phagosome, Type I diabetes mellitus et al. **Figure S9.** The KEGG analysis of hypomethylated genes. The most enriched KEGG targets were involved in Type I diabetes mellitus, allograft rejection, graft-versus-host disease et al. **Figure S10.** SDR42E1 CpG derived methylation data**. Figure S11.** CAMTA1 CpG derived methylation data. **Figure S12.** TSTD1 CpG derived methylation data. **Figure S13.** PIP5K1C CpG derived methylation data. **Figure S14.** DNER CpG derived methylation data. **Figure S15.** KIAA1614 CpG derived methylation data.

## Data Availability

All data generated or analyzed during this study are included in this published article.

## Abbreviations

hEP: Human Ectopic Pregnancy
5-mC: 5-methylcytosine
DNMTs: DNA methyltransferase
CpG: Cytosine Guanine
SWAN: Subset Within-Array Normalization
BSP: Bisulfite Sequencing PCR
C: Cytosine
T: Thymine
U: Uracil
GO: Gene Ontology
KEGG: Kyoto Encyclopedia of Genes and Genomes
PPI: Protein Protein Interaction
TLN: Talins
TST: Sulfurtransferase
GSS: S-sulfanylglutathione
SQOR: Sulfide, quinone oxidoreductase
SDO: Sulfur dioxygenase
WGCNA: Weighted Correlation Network Analysis
DEGs: Different Expression Gene set
