# Supplementary material for: Abnormally increased DNA methylation in chorionic tissue might play an important role in development of ectopic pregnancy
Source: Reprod Biol Endocrinol. 2021 Jul 2;19:101. doi: 10.1186/s12958-021-00785-2 (PMC8252306; doi:10.1186/s12958-021-00785-2)
Supplement: Supplementary file 1 — Additional file 1: Figure S1. T-test of all differential methylated genes. Figure S2. The biological process GO analysis of hypermethylated genes. The most enriched GO targets were involved in T-helper 1 type immune response, homophilic cell adhesion via plasma membrane adhesion molecules, positive regulation of filopodium assembly et al. Figure S3. The biological process GO analysis of hypomethylated genes. The most enriched GO targets were involved in homophilic cell adhesion via plasma membrane adhesion molecules, transcription from RNA polymerase II promoter, nervous system development et al. Figure S4. The cellular component GO analysis of hypermethylated genes. The most enriched GO targets were involved in transport vesicle membrane, clathrin-coated endocytic vesicle membrane, trans-Golgi network membrane et al. Figure S5. The cellular component GO analysis of hypomethylated genes. The most enriched GO targets were involved in plasma membrane, integral component of plasma membrane, MHC class II protein complex et al. Figure S6. The molecular function GO analysis of hypermethylated genes. The most enriched GO targets were involved in phosphatidylinositol-3-phosphate binding, GTPase activator activity, calcium ion binding et al. Figure S7. The molecular function GO analysis of hypomethylated genes. The most enriched GO targets were involved in calcium binding, sequence-specific DNA binding, RNA polymerase II core promoter proximal region sequence-specific DNA binding et al. Figure S8. The KEGG analysis of hypermethylated genes. The most enriched KEGG targets were involved in leishmaniasis, phagosome, Type I diabetes mellitus et al. Figure S9. The KEGG analysis of hypomethylated genes. The most enriched KEGG targets were involved in Type I diabetes mellitus, allograft rejection, graft-versus-host disease et al. Figure S10. SDR42E1 CpG derived methylation data. Figure S11. CAMTA1 CpG derived methylation data. Figure S12. TSTD1 CpG derived methylation data. Figure [file 12958_2021_785_MOESM1_ESM.docx]

**Supplementary material**

**Abnormally increased methylation in chorionic tissue might play an important role in development of ectopic pregnancy**

Wen Cai^1^, Liu Yang^1^, Ruiqing Zhang, Yixia Yang, Shungdi Li*, Jiarong Zhang*

**Figure S1 T-test of all differential methylated genes**


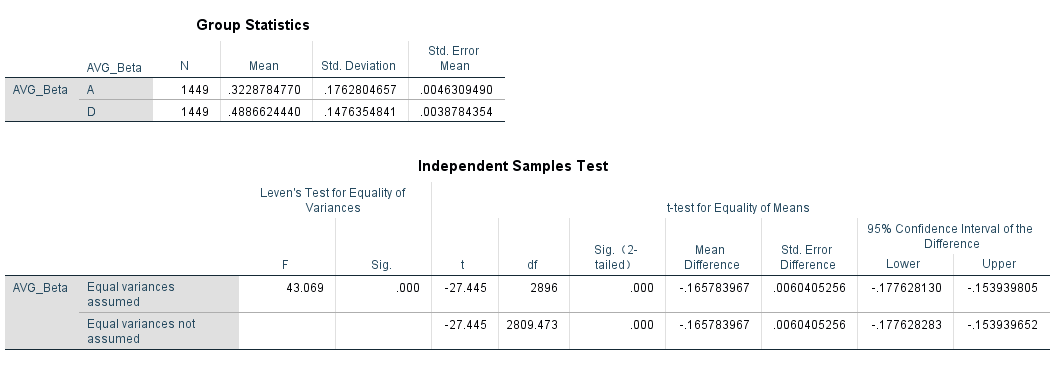


**Fig.****S1:** T-test of all differential methylated genes

**Figure S2** **The biological process GO analysis of hypermethylated genes**

**
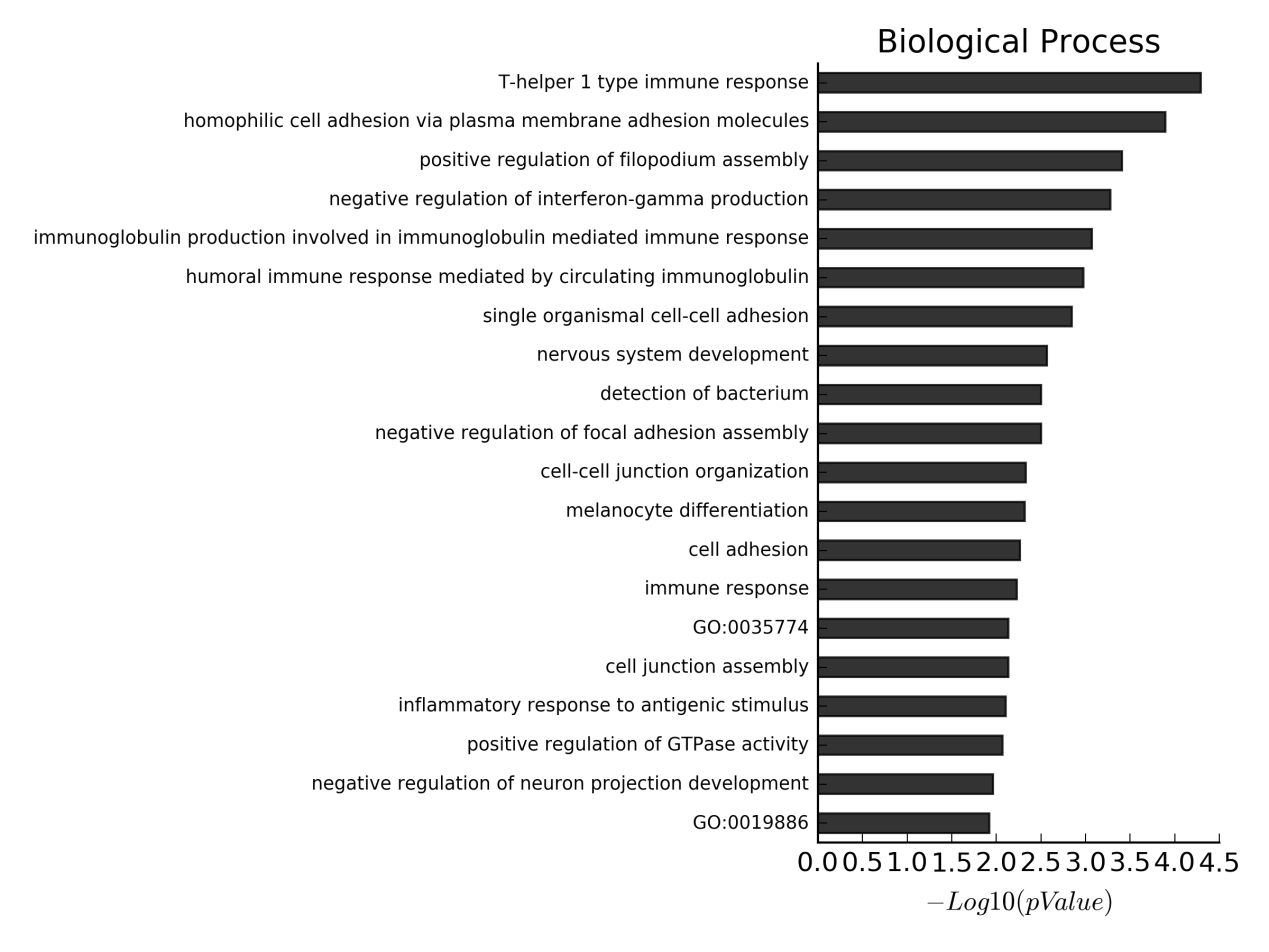
**

**Fig.S2:** The most enriched GO targets were involved in T-helper 1 type immune response, homophilic cell adhesion via plasma membrane adhesion molecules, positive regulation of filopodium assembly et al.

**Figure S3 The biological process GO analysis of hypomethylated genes**

**
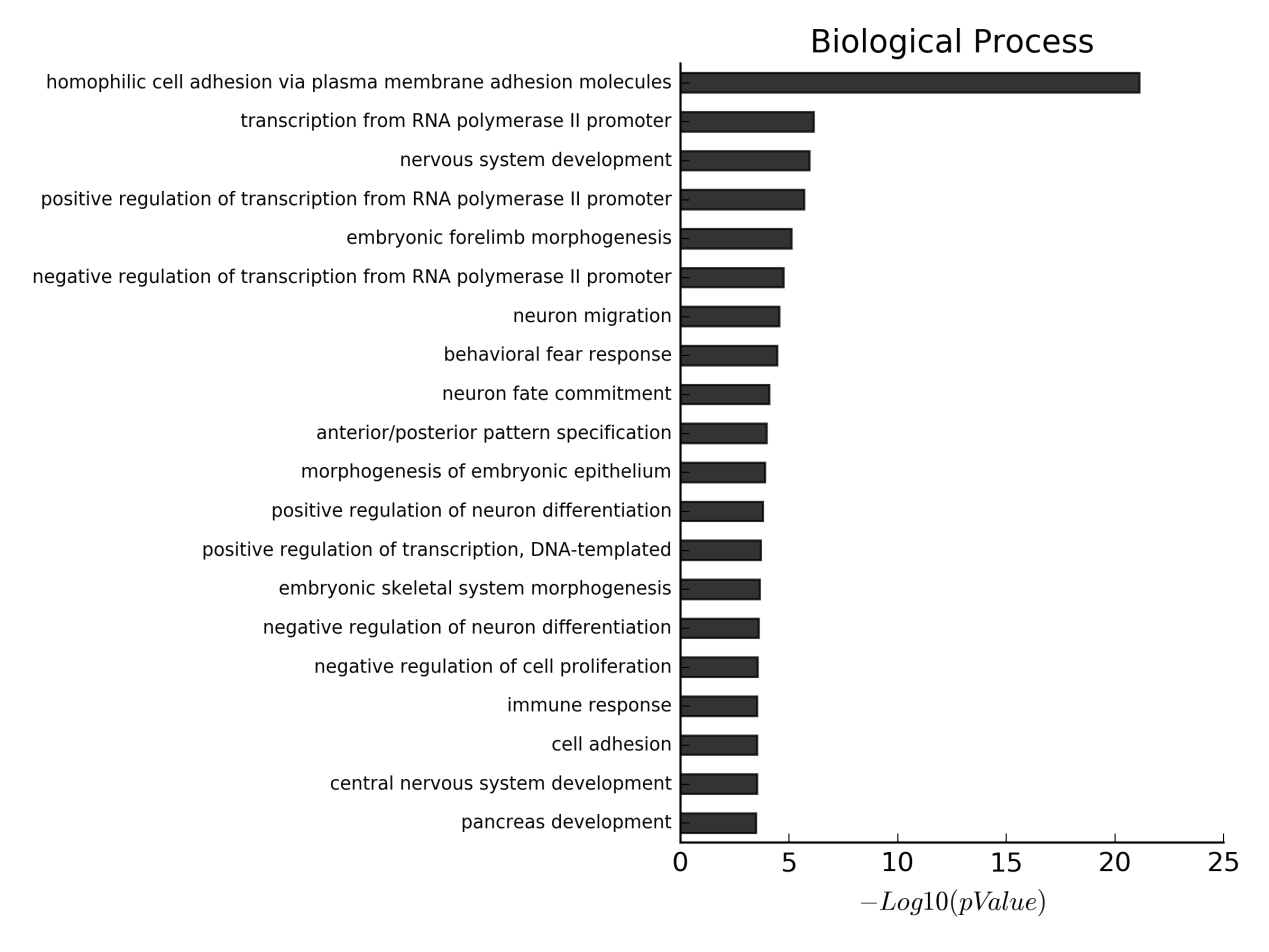
**

**Fig.S3:** The most enriched GO targets were involved in homophilic cell adhesion via plasma membrane adhesion molecules, transcription from RNA polymerase II promoter, nervous system development et al.

**Figure S4 The cellular component GO analysis of hypermethylated genes**


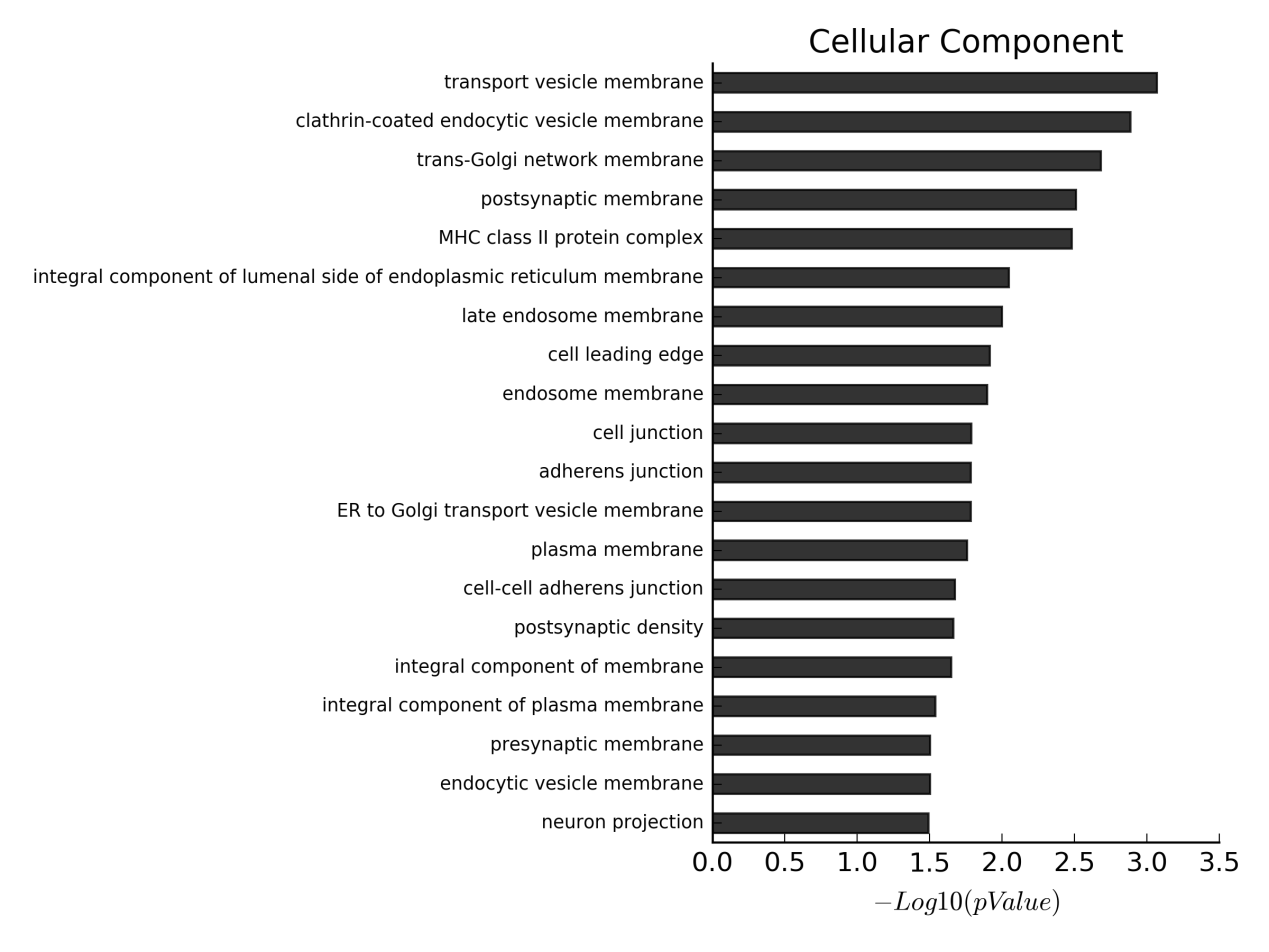


**Fig.S4:** The most enriched GO targets were involved in transport vesicle membrane, clathrin-coated endocytic vesicle membrane, trans-Golgi network membrane et al.

**Figure S5 The cellular component GO analysis of hypomethylated genes**

**
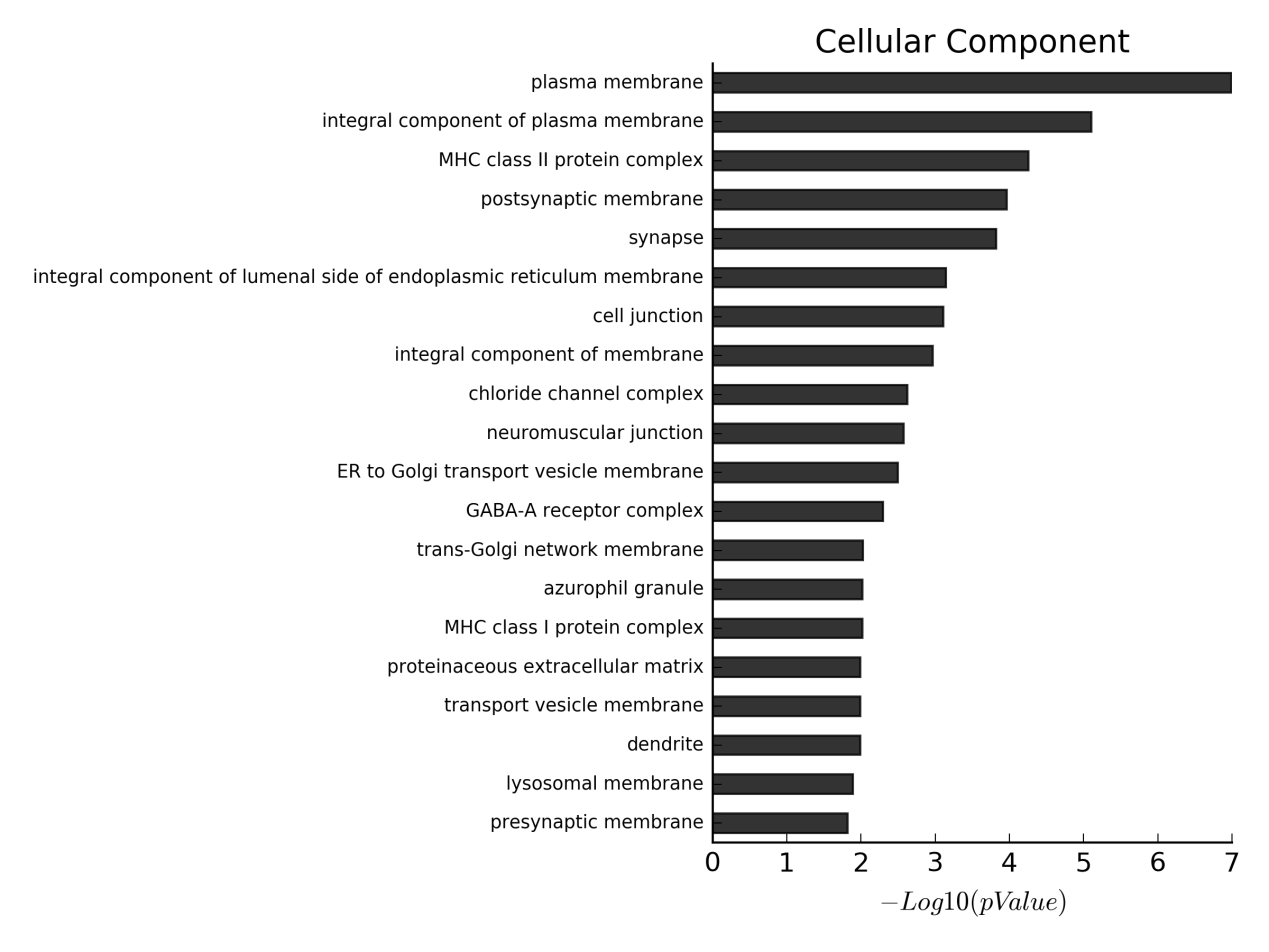
**

**Fig.S5:** The most enriched GO targets were involved in plasma membrane, integral component of plasma membrane, MHC class II protein complex et al.

**Figure S6 The molecular function GO analysis of hypermethylated genes**


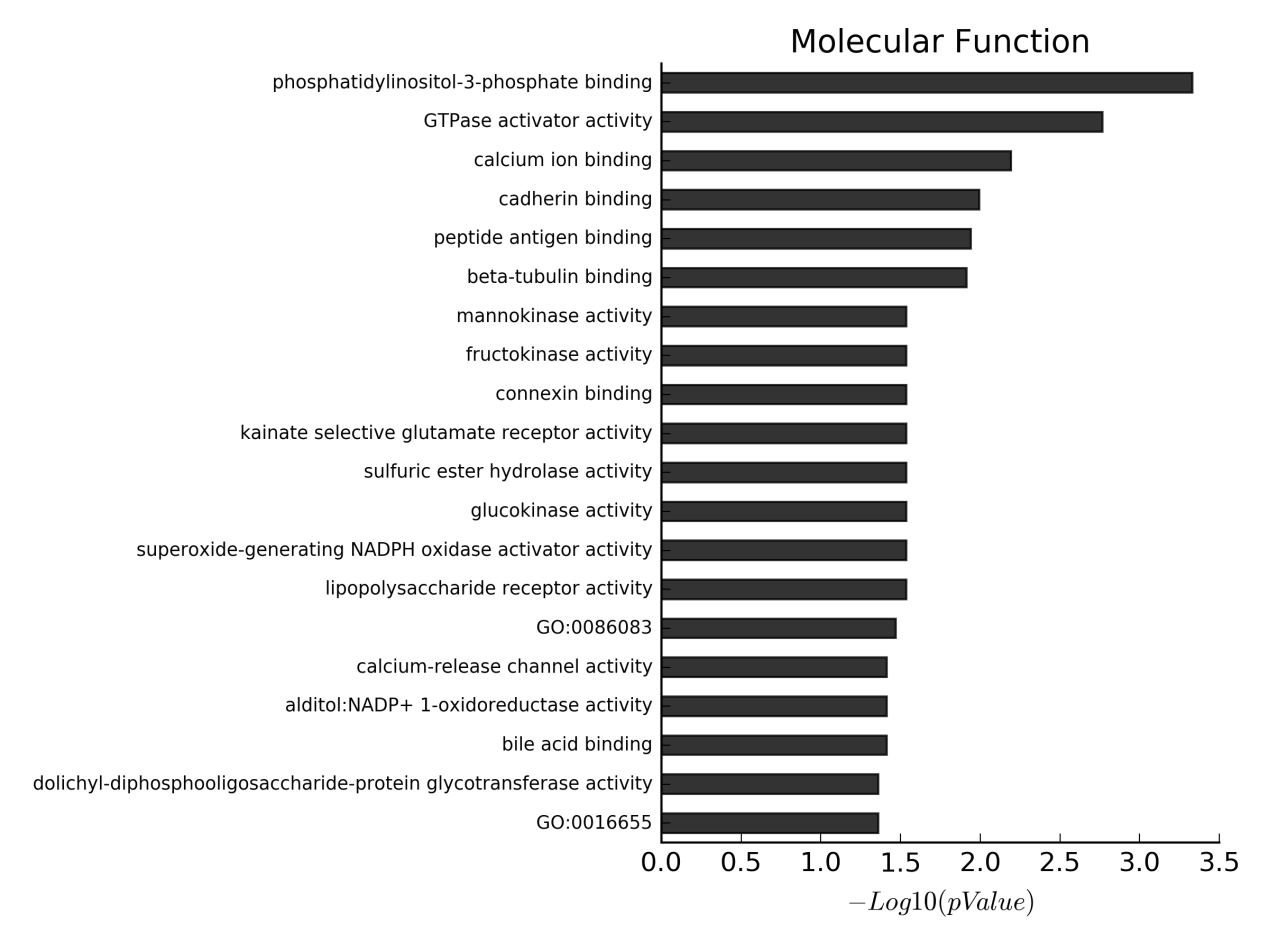


**Fig.S6:** The most enriched GO targets were involved in phosphatidylinositol-3-phosphate binding, GTPase activator activity, calcium ion binding et al.

**Figure S7 The molecular function GO analysis of hypomethylated genes**


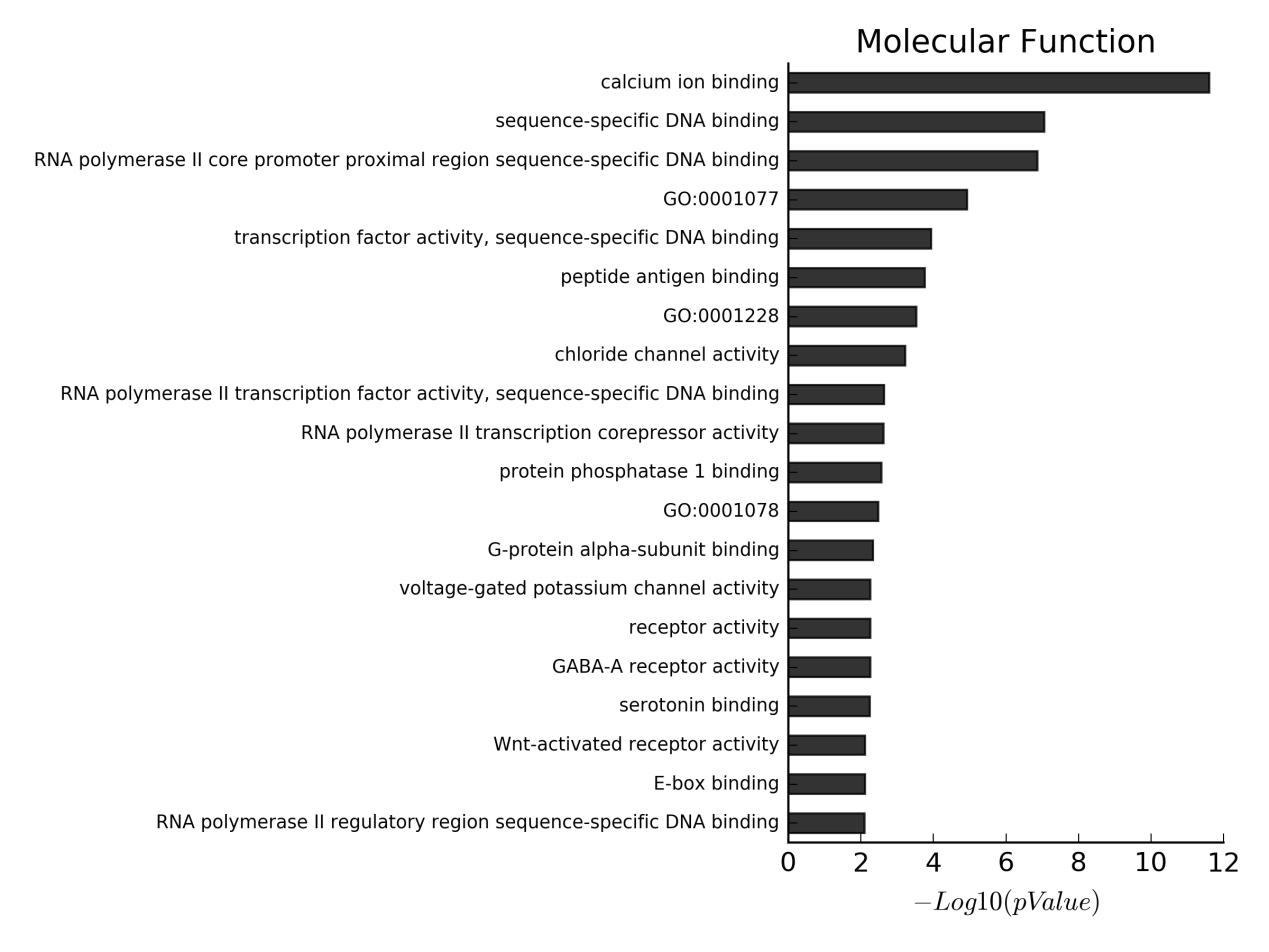
**Fig.S7:** The most enriched GO targets were involved in calcium binding, sequence-specific DNA binding, RNA polymerase II core promoter proximal region sequence-specific DNA binding et al.

**Figure S8 The KEGG analysis of hypermethylated genes**


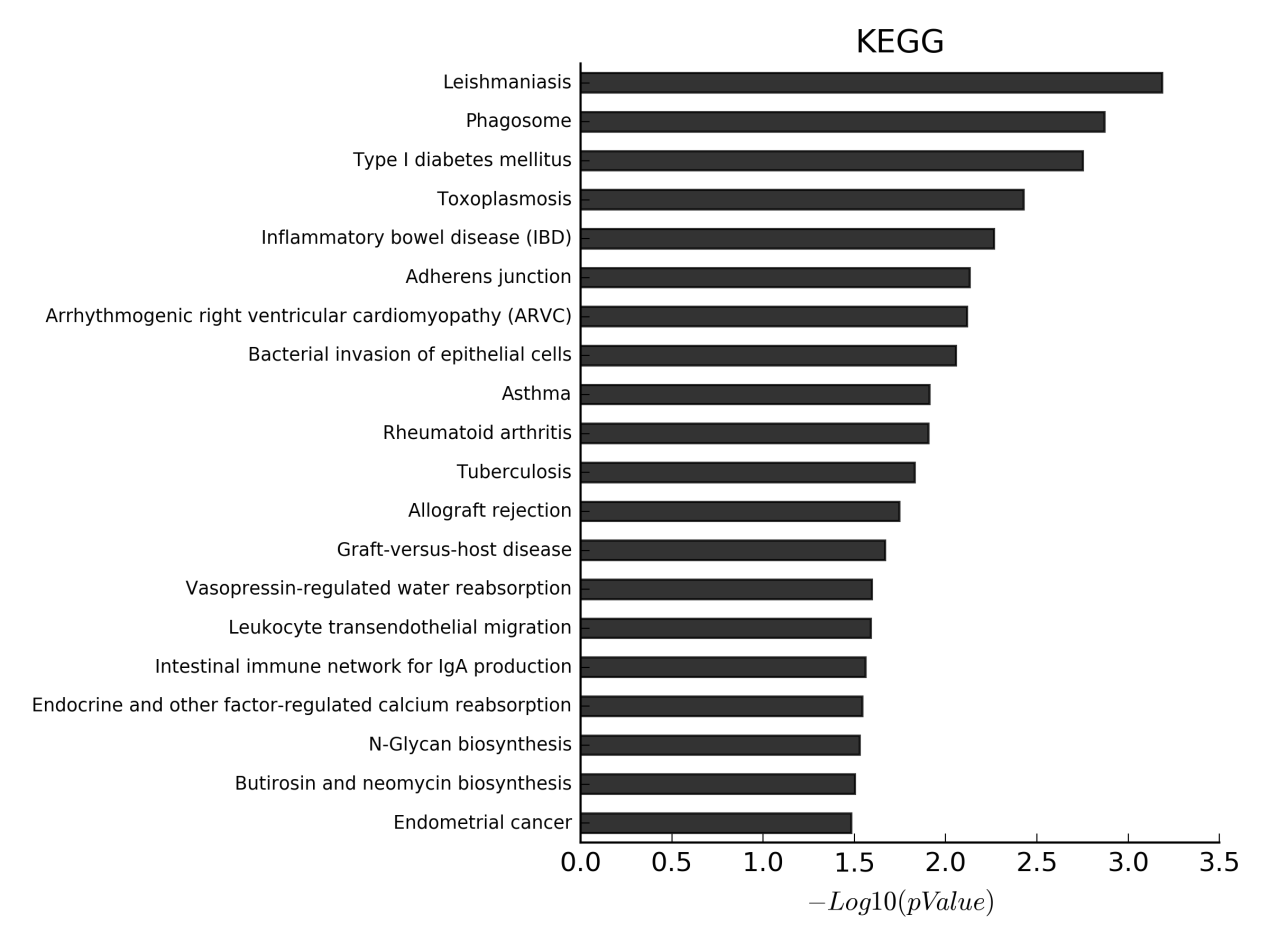
**Fig.S8:** The most enriched KEGG targets were involved in leishmaniasis, phagosome, Type I diabetes mellitus et al.

**Figure S9 The KEGG analysis of hypomethylated genes**

**
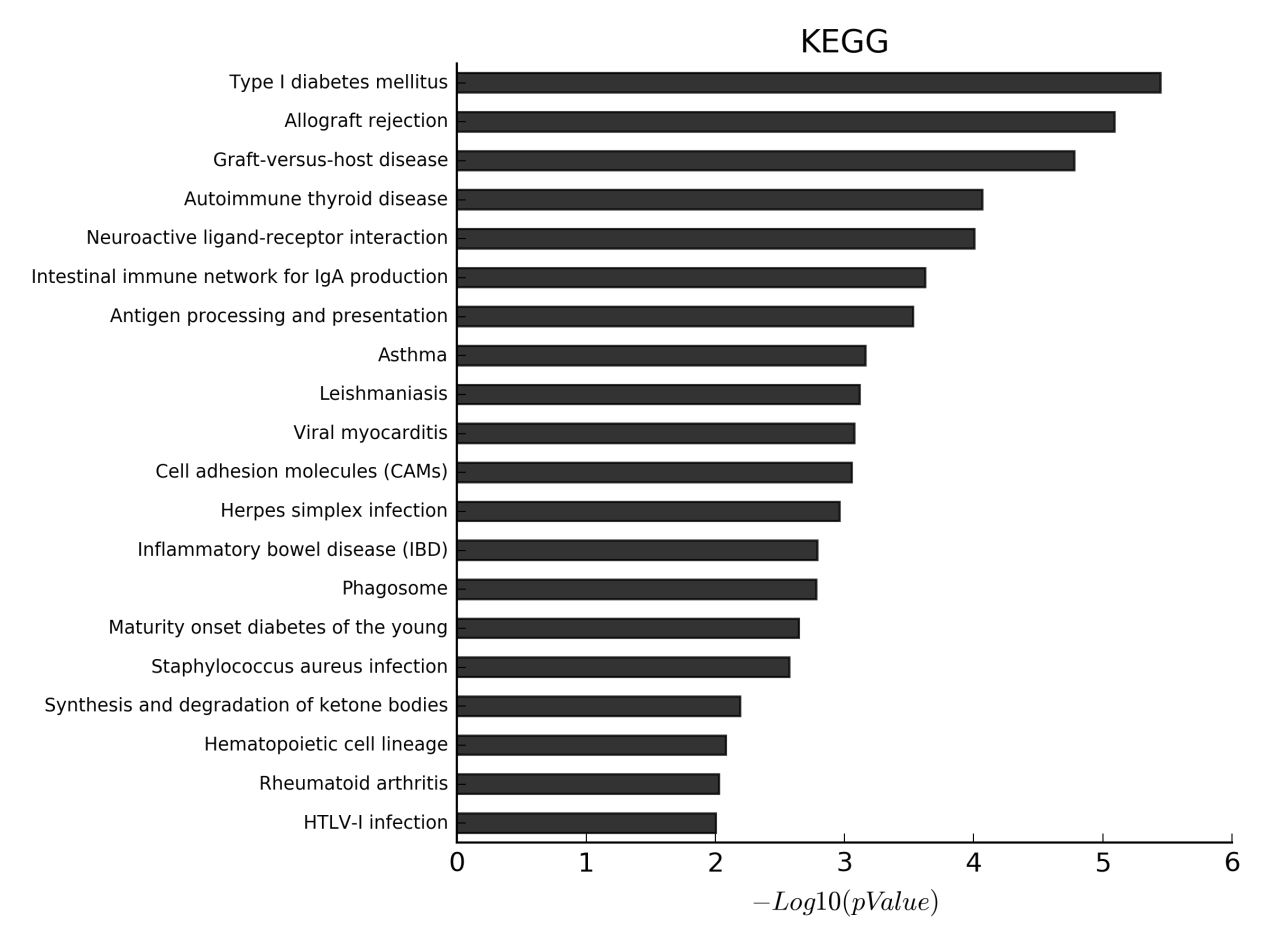
**

**Fig.S9:** The most enriched KEGG targets were involved in Type I diabetes mellitus, allograft rejection, graft-versus-host disease et al.

**Figure S10 SDR42E1 CpG derived methylation data**

**
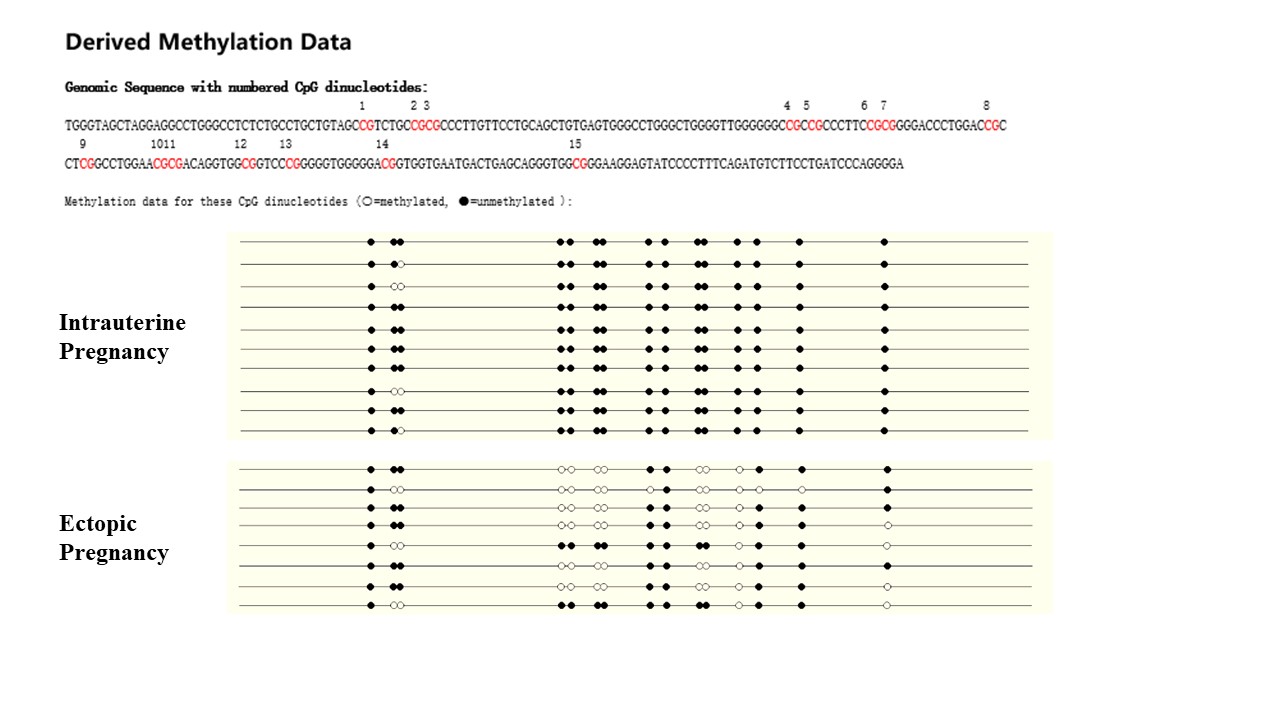
**

**Fig.S10:** SDR42E1 CpG derived methylation data

**Figure S11 CAMTA1 CpG derived methylation data**

**
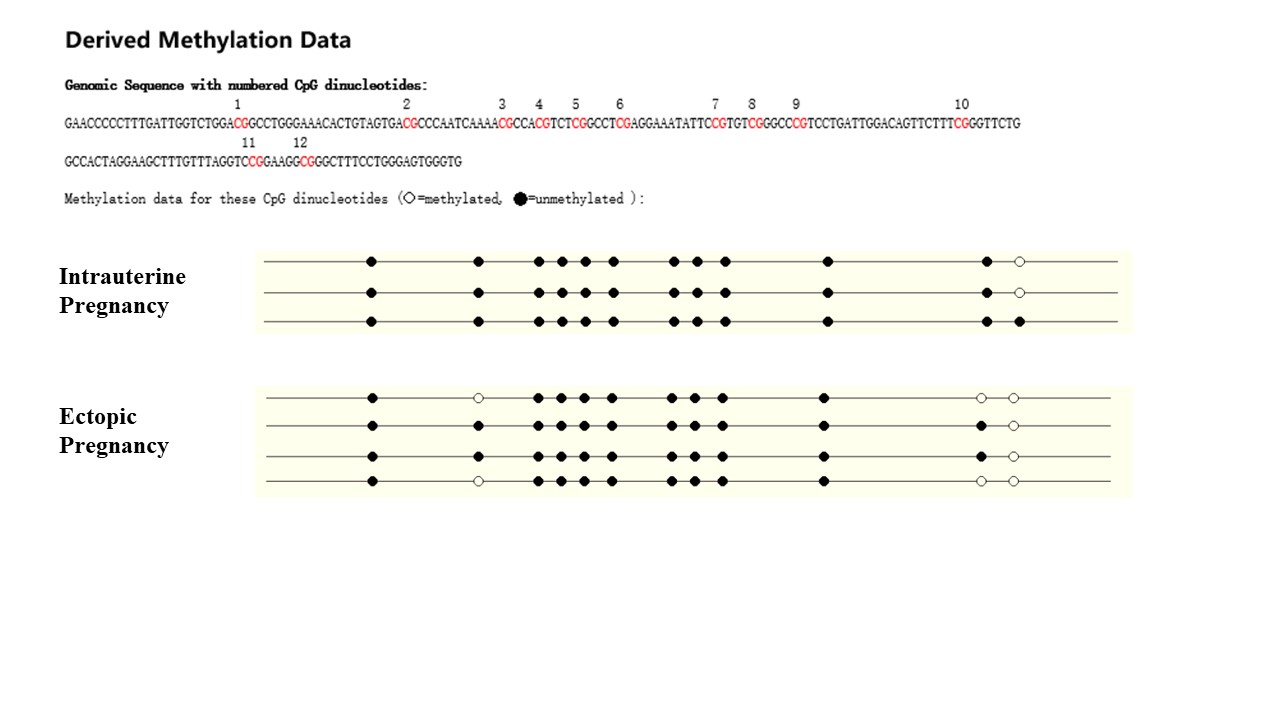
**

**Fig.S11:** CAMTA1 CpG derived methylation data

**Figure S12 TSTD1 CpG derived methylation data**

**
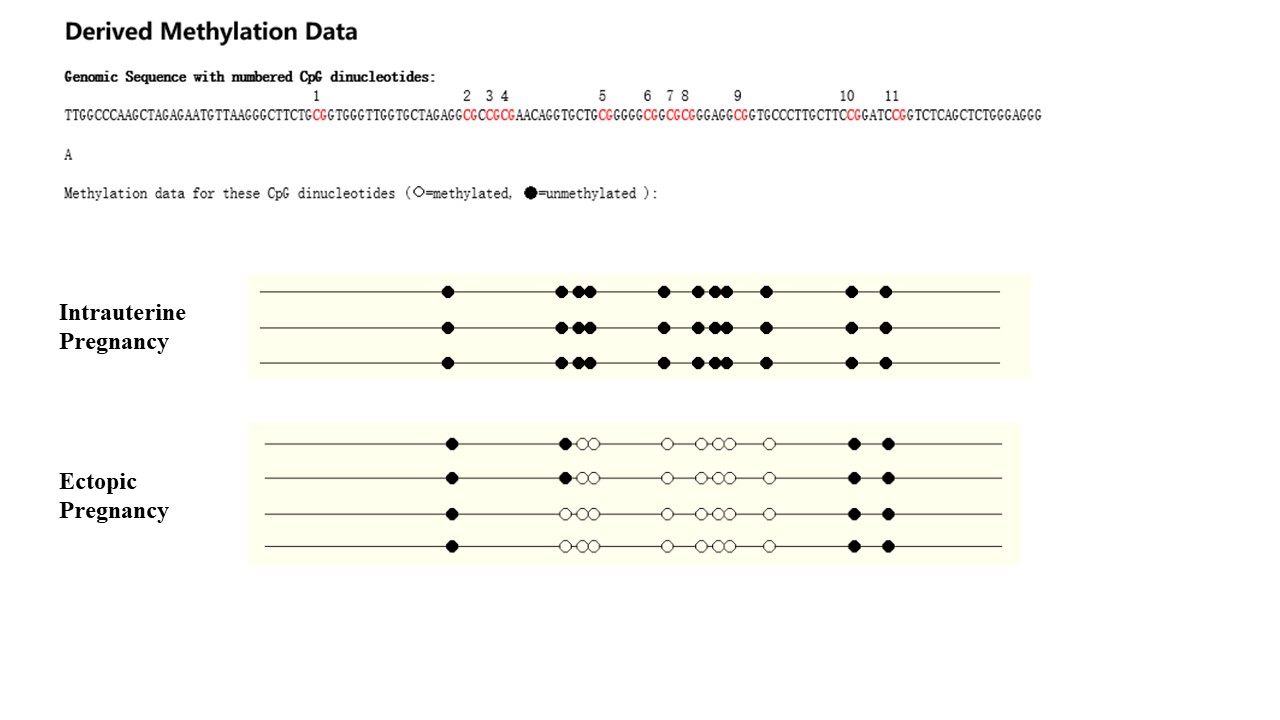
**

**Fig.S12:** TSTD1 CpG derived methylation data

**Figure S13 PIP5K1C CpG derived methylation data**

**
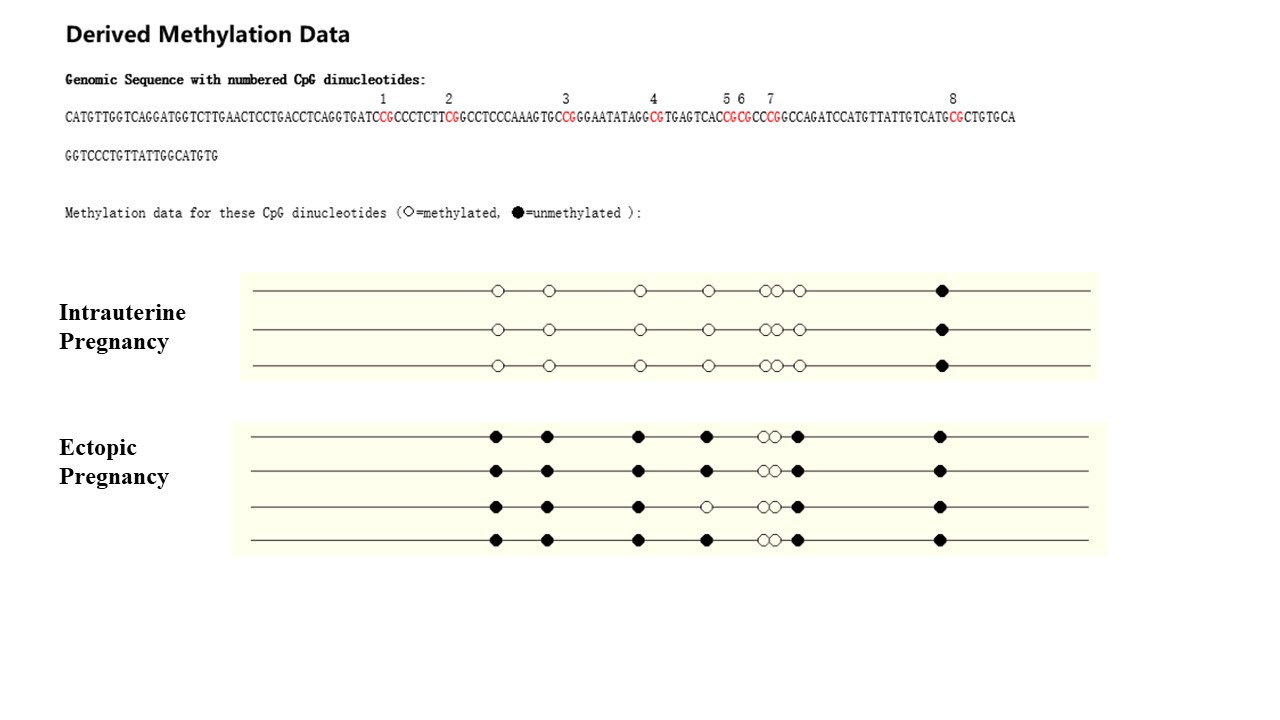
**

**Fig.S13:** PIP5K1C CpG derived methylation data

**Figure S14 DNER CpG derived methylation data**

**
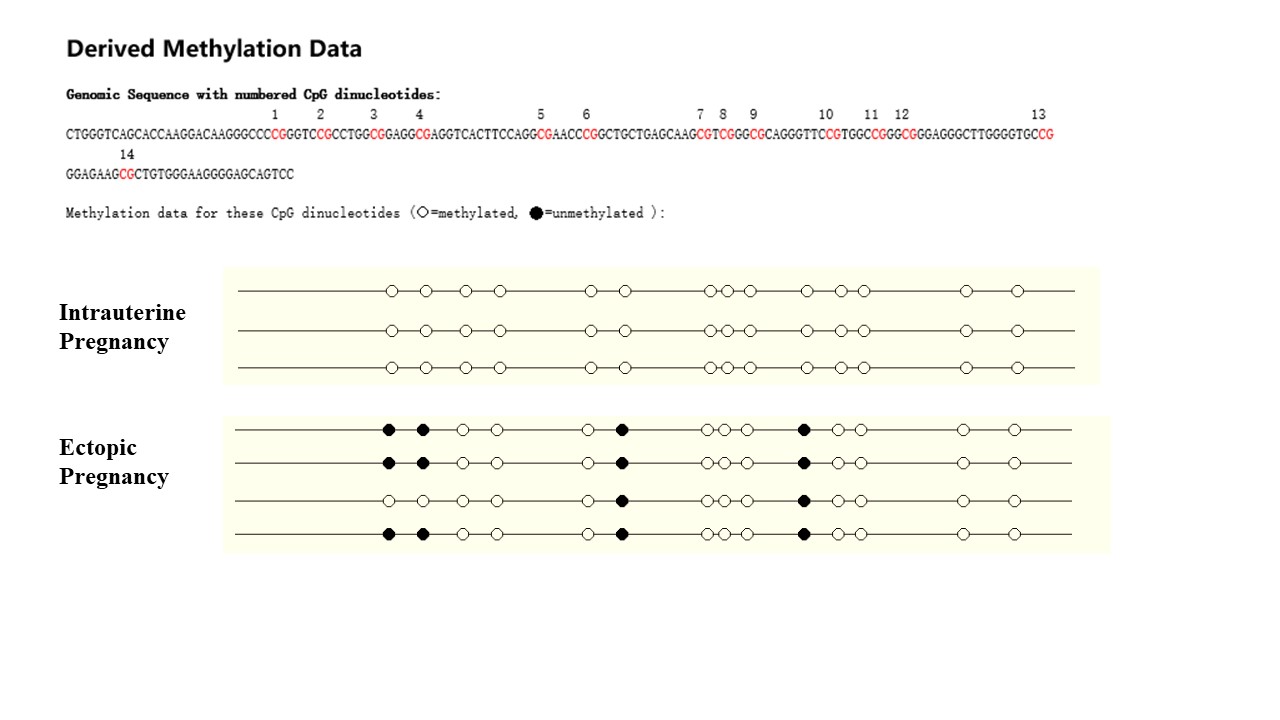
**

**Fig.S14:** DNER CpG derived methylation data

**Figure S15 KIAA1614 CpG derived methylation data**

**
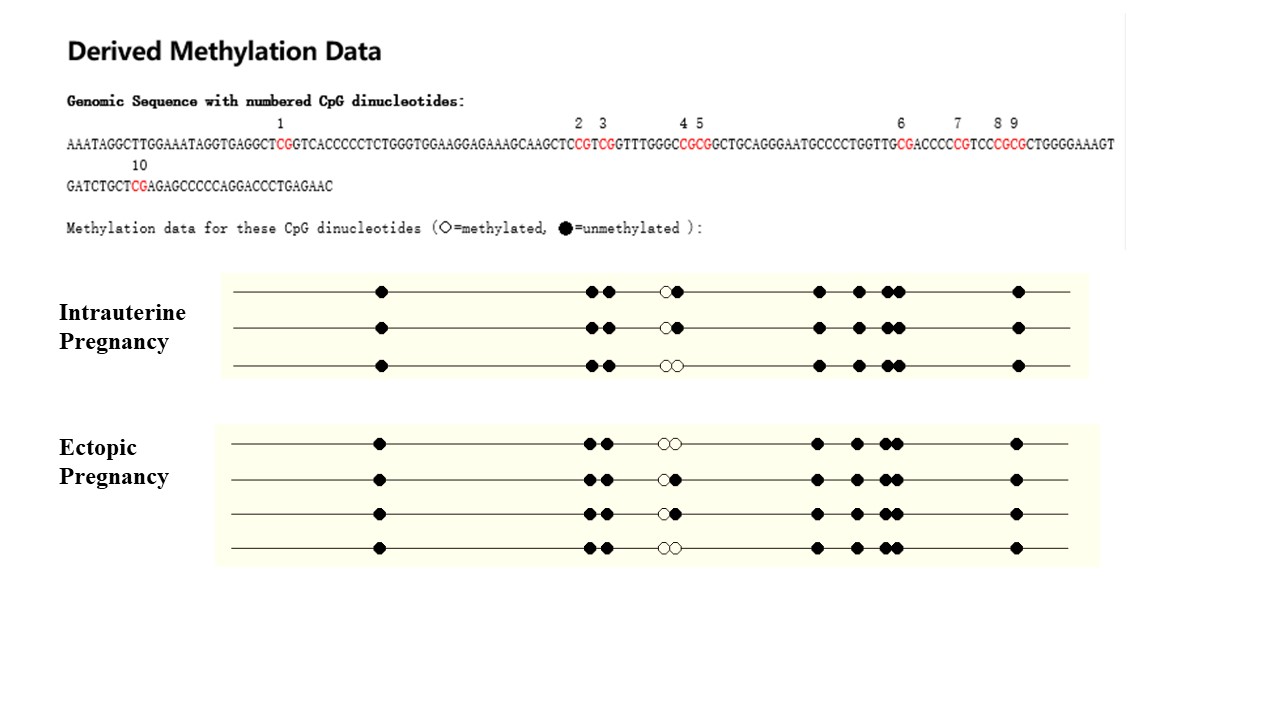
**

**Fig.S15:** KIAA1614 CpG derived methylation data
